# The Association of Lesion Location and Sleep Related Breathing Disorder in Patients with Acute Ischemic Stroke

**DOI:** 10.1371/journal.pone.0171243

**Published:** 2017-01-30

**Authors:** Anna Lena Fisse, André Kemmling, Anja Teuber, Heike Wersching, Peter Young, Ralf Dittrich, Martin Ritter, Rainer Dziewas, Jens Minnerup

**Affiliations:** 1 Department of Neurology, University of Muenster, Muenster, Germany; 2 Department of Neuroradiology, University of Luebeck, Luebeck, Germany; 3 Institute of Epidemiology and Social Medicine, University of Muenster, Muenster, Germany; 4 Department of Sleep Medicine and Neuromuscular Disorders, Muenster, Germany; University Hospital-Eppendorf, GERMANY

## Abstract

**Background and aims:**

Sleep related breathing disorders (SRBD) are common in patients with ischemic stroke and are associated with poor outcome. SRBD after stroke were assumed to be a direct consequence of injury of specific central nervous system structures. However, whether specific locations of ischemic infarcts cause SRBD is yet unknown. We therefore investigated the association of ischemic lesion location with SRBD.

**Methods:**

Patients with acute ischemic stroke treated on our stroke unit were included in a prospective observational study. All patients underwent magnetic resonance imaging (MRI) and polygraphy in the acute phase after stroke. SRBD was defined by an apnea—hypopnea index (AHI) ≥10. MRI were evaluated using standardized maps to depict voxel-wise probability distribution of infarction for patients with and without SRBD. Groups were compared using logistic regression analysis.

**Results:**

Of 142 patients included, 86 (59%) had a SRBD. Age, body mass index and prevalence of arterial hypertension were significantly higher in patients with SRBD. There was no statistically significant association between any lesion location and SRBD.

**Conclusion:**

We found no association of lesion location and SRBD in stroke patients, whereas established risk factors for SRBD, known from general population, were significantly associated with SRBD. Given the high prevalence of SRBD in stroke patients, these findings suggest that cerebral ischemia facilitates the occurrence of SRBD in patients with pre-existing risk factors rather than causing it by damaging specific central nervous system structures. Our findings can be used to identify stroke patients who might benefit from polygraphy screening.

## Introduction

Sleep related breathing disorders (SRBD) are common in patients with stroke, occurring in more than half of all patients after stroke [1] and significantly affect the clinical outcome [2]. The prevalence of SRBD is increased in patients with ischemic as well as haemorrhagic stroke [3]. In the general population established risk factors for SRBD are older age, male sex and obesity [4]. In patients with stroke, the relationship between SRBD and stroke is not clear yet. SRBD is assumed to be both, a risk factor for stroke and a complication following stroke [5]. In the latter case, SRBD after stroke has been discussed to be a direct consequence of the injury of specific central nervous system structures [6],[7]. Particularly brainstem infarctions have previously been suggested to be associated with SRBD after stroke, as they can impair the regulation of breathing and the tone of the upper airways [6]. However, conclusive results regarding the association of SRBD and ischemic lesion location are lacking.

### Aims

The aim of this study was to investigate whether the diagnosis of SRBD in patients with acute ischemic stroke is associated with specific lesion locations.

## Methods

The study protocol was approved by the local ethics committee of the University of Munster and written informed consent was obtained from each patient.

### Patients

Overall, 142 patients with acute ischemic stroke admitted to the stroke unit of the Department of Neurology of the University Hospital Muenster, Germany, were included in a prospective observational study during the study period from December 2001 to February 2004. Baseline demographic variables, medical history and parameters on the present stroke were recorded. Patients underwent magnetic resonance imaging (MRI) and polygraphy in the acute phase after stroke. Patients with severely decreased consciousness and unstable medical conditions such as severe infection or decompensated congestive heart failure were excluded.

### Clinical assessment

The severity of stroke was assessed on admission using the National Institutes of Health Stroke Scale (NIHSS) [8]. The aetiology of stroke was rated according to the modified TOAST criteria [9]. The severity of disability was determined using the Modified Rankin Scale. Cerebrovascular risk factors were acquired in all patients, including body mass index (BMI), smoking habits (active smokers vs. non-smokers; ex-smokers were considered non-smokers when they had stopped smoking more than 3 months prior to the stroke), hypercholesterolemia (history of hypercholesterolemia and/or fasting total cholesterol level over 200 mg/dl), arterial hypertension (history of hypertension and/or measured systolic blood pressure above 140 mmHg and/or diastolic blood pressure above 90 mmHg on the second day of admission), and diabetes mellitus (history of diabetes mellitus and/or measured fasting blood glucose over 126 mg/dl).

### Polygraphy

Cardiorespiratory polygraphy was performed with a portable cardiorespiratory recording device (Merlin, Heinen and Löwenstein, Bad Ems, Germany) during the first 72h after admission. This device measures nasal and oral airflow, chest and abdominal movements, body position, heart rate and oxygen saturation. Measurement started at 10 p.m. and ended at 6 a.m. on the next morning. Apnea was defined as the cessation of airflow for at least 10 seconds. Hypopnea was considered a thoracoabdominal amplitude decrease of > 50% for at least 10 seconds. The apnea-hypopnea index (AHI) was defined as the average number of apneas and hypopneas per hour of sleep. SRBD was defined as AHI ≥10.

### Imaging and image analysis

Following acute stroke triage, all patients underwent cerebral MRI on follow-up within 12 days including diffusion-weighted imaging (DWI) and FLAIR (1.5 Tesla Intera Gyroscan, Philips, Best, The Netherlands; DWI with TR 4.25ms, TE 95ms, matrix 256x256, FOV 230x230 mm, transversal 5mm thick slices, b-values 0 and 1.0 mm^2/s; FLAIR with TR 8.00ms, TE 120ms, transversal 5mm thick slices).

Final infarct lesions were manually segmented slice by slice on the individual MRI scans (Analyze 10.0, AnalyzeDirect Inc., Overland Park, USA). Original images and corresponding binary lesion masks were affine registered to standard MNI-152 brain (FLIRT, FSL 5.0,FMRIB Software Library, Oxford, UK) optimized by non-linear refinement (FNIRT, FSL 5.0) using high precision modality specific population based reference images [10]. Lesion volumes were normalized to head size. Atlas based analysis of brain infarct was performed. The percentage of a brain region that was infarcted was calculated for each patient using the Harvard-Oxford cortical and subcortical structural atlases and the JHU white-matter tractography atlas (137 regions in total) implemented in the FMRIB Software Library [11]. Regions with less than 1% of infarcted voxels were excluded from analysis.

### Statistical analysis

Patient groups (SRBD vs. non-SRBD) were compared using Student’s t test (normal distribution) and Mann—Whitney U test (non-normal distribution) for quantitative variables and chi-squared test for categorical variables including lesion location. Bonferroni-Holm correction was used to adjust for multiple comparisons (137 ROIs). For every ROI that showed a statistical significant association with SRBD in the univariate analysis a logistic regression analysis was used to adjust for age, sex and all covariates showing significance at p < 0.05 in a single-factor analysis. In addition, a logistic regression analysis was performed to evaluate whether covariates were independently associated with SRBD. The level of significance was defined as p < 0.05 and according to Bonferroni Holm correction, respectively. Statistical analyses were performed using IBM SPSS Statistics Version 22.0.0.0.

## Results

### Baseline data

Of 142 patients, 86 (59%) had a SRBD. Main characteristics of patients are summarized in Table 1. Patients with SRBD were significantly older, had a higher BMI and were more often classified as hypertensive compared to patients without SRBD. The distribution of sex as well as NIHSS and modified Rankin Score did not differ between both groups. In a multivariate analysis only a higher age was significantly associated to the occurrence of SRBD (p<0.01). Stroke aetiology differed in both groups: atherosclerosis was more frequent in SRBD patients and cardioembolism was more frequent in patients without SRBD.

**Table 1:** Baseline characteristics of patients with and without sleep related breathing disorder (SRBD).

	SRBD (n = 86)	non-SRBD (n = 56)	p
Demographics			
Age, mean (SD), y	68 (±10)	60 (±17)	<0.001
Women, n (%)	28 (32.6)	21 (37.5)	0,59
BMI, mean (SD), kg/m^2^	27 (±4.3)	25.4 (±3.6)	0,04
Comorbidities [n], (%)			
Hypertension	77 (89.5)	39 (69.6)	<0.001
Diabetes mellitus	26 (30.2)	10 (17.9)	0,12
Hyperlipidemia	37 (43)	27 (48.2)	0,61
Smoker	26 (30.2)	21 (37.5)	0,47
Stroke aetiology [n], (%)			
Atherosclerosis	39 (45)	16 (29)	0,05
Cardioembolism	25 (29)	27 (48)	0,02
Lacunar stroke	12 (14)	6 (11)	0,57
Other determined aetiology	2 (2)	3 (5)	0,38
Undetermined aetiology	8 (9)	4 (7)	0,76
NIHSS score, median (IQR)	6 (7)	5.5 (8)	0,3
Modified Rankin scale, median (IQR)	3 (2)	3 (2)	0,68

SRBD, sleep related breathing disorder; BMI, body mass index; NIHSS, National Institutes of Health Stroke Scale; SD, standard deviation; IQR, interquartile range.

### Association of lesion location and sleep related breathing disorder

**Fig 1:**
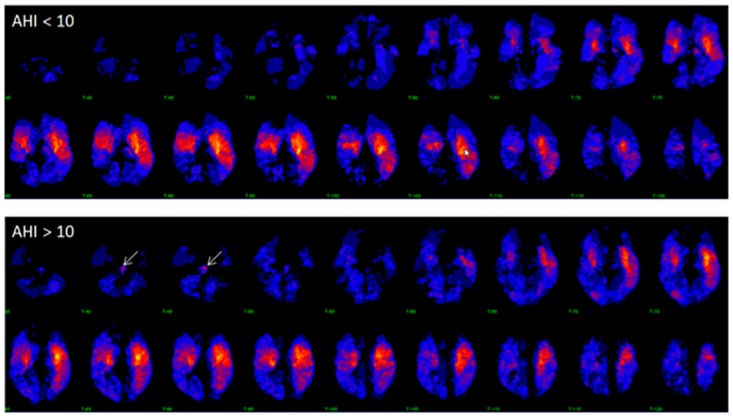
The distribution of infarctions for patients with (AHI > 10) and without (AHI < 10) SRBD. Blue shades: less frequent infarcted regions; Red shades: more frequent infarcted regions. Brainstem infarcts appear to be more frequent in patients with SRBD (arrow, not significant).

The distribution of infarctions for patients with and without SRBD is shown in Fig 1. None of the 137 predefined regions showed a statistically significant difference in infarct frequency between patients with and without SRBD. The voxel-based probability map of infarcts suggested that brainstem infarcts are more frequent in patients with SRBD than without SRBD (Fig 1). Among patients with SRBD, 10 of 86 (11.6%) and among patients without SRBD, 2 of 56 (3.6%) had a brainstem infarct. However, this difference was statistically not significant (p = 0.09).

## Discussion

In our cohort of stroke patients 59% had a SRBD. Higher age was significantly associated with SRBD, whereas no specific lesion location was related to the occurrence of SRBD.

In accordance with prior findings [6],[7], we showed that SRBD is a frequent disorder after stroke. However, results on the association between SRBD and infarct location were conflicting in previous studies [7]. Especially infarcts within the brainstem were hypothesised to cause post-stroke SRBD, as they could impair the regulation of breathing and the tone of the upper airways [6]. In a previous study with patients with acute lacunar stroke, lesions located in the internal capsule and the pons were associated with SRBD [12]. In a large population based study of 355 patients an acute infarction of the brainstem was associated with the presence and higher severity of SRBD in an unadjusted analysis, which dissolved after adjustment for cerebrovascular risk factors and stroke severity [6]. A large prospective Korean study (n = 293) and an U.S. study among 73 patients also failed to show a correlation between lesion location and SRBD [7,13]. Some authors stated that the typical risk factors for SRBD in the general population are not predictive of SRBD in the acute stroke population [7]. However, in our study we found classical risk factors to be associated with SRBD after stroke. This finding in combination with the high frequency of SRBD after stroke suggests that stroke facilitates the occurrence of SRBD in already susceptible patients rather than causing SRBD due to a specific lesion pattern. From a clinical perspective, this implies that polygraphy for the detection of post-stroke SRBD should particularly be used when additional risk factors are present.

This study has strengths and limitations. Manual lesion delineation and voxel based lesion-symptom mapping is an excellent method as it allows the exact investigation of tissue damage and related clinical feature on a voxel-by-voxel basis [14]. In the majority of previous studies investigating SRBD and lesion location, it was examined whether specific brain regions were infarcted or not, the relative size of the lesions was not measured. According to our present knowledge, our study is the first to analyse the lesion size voxel-wise. In addition, polygraphy was performed in the acute phase of stroke, therefore early evaluation of the impact of infarction on SRBD was possible. SRBD is more severe in the acute phase after stroke and improves during recovery. A meta-analysis of 29 studies showed that within the first 7 days after stroke more than 60% of patients had SRBD with an AHI>10. 53% of patients still exhibited an AHI >10 after 4 weeks [15]. SRBD is associated with early neurological worsening after stroke. The assumed mechanism is sleep apnea-induced hypercapnia with subsequent cerebral vasodilatation aside the ischemic area and steal of blood from ischemic areas [16]. Therefore, polygraphy within the first 72 hours presumable covers the clinically meaningful time period.

A general limitation of studies on post-stroke SRBD is that SRBD developed after stroke cannot be distinguished from SRBD existing prior to stroke. Another limitation is, that we cannot distinguish between the different types of SRBD in this study. Furthermore, excluding severely ill patients, as performing polygraphy in intubated or dyspnoeic patients is not possible, might be a potential source of bias. However, this may have the advantage that the lesions do not involve the whole hemisphere, and therefore specific regions related to SRBD could be investigated better. Studies with neutral results have a potential general weakness, i.e., the type II error. The type II error means that the null hypothesis is not rejected despite being false. We therefore cannot rule out an association of lesion location and SRBD with absolute certainty.

## Conclusions

In this study, we found no association of lesion location and the occurrence of SRBD. Known risk factors for SRBD in the general population, i.e. age, were significantly associated with post-stroke SRBD. We conclude that post-stroke SRBD rather is the result of an accumulation of multiple risk factors in addition to the stroke itself, and not the result of an infarction in a specific location. Given these findings, SRBD should be assessed particularly in older stroke patients with atherosclerotic stroke etiology. Our findings should be considered for the identification of stroke patients screened with polygraphy and in further studies targeting post-stroke SRBD.
